# Demonstration of infectious salmon anaemia virus (ISAV) endocytosis in erythrocytes of Atlantic salmon

**DOI:** 10.1186/1743-422X-4-13

**Published:** 2007-01-25

**Authors:** Samuel T Workenhe, Dorota W Wadowska, Glenda M Wright, Molly JT Kibenge, Frederick SB Kibenge

**Affiliations:** 1 Department of Pathology and Microbiology, Atlantic Veterinary College, University of Prince Edward Island, 550 University Avenue, Charlottetown, PE. C1A 4P3. Canada; 2 Electron Microscopy Laboratory, Atlantic Veterinary College, University of Prince Edward Island, 550 University Avenue, Charlottetown, PE. C1A 4P3. Canada; 3 Department of Biomedical Sciences, Atlantic Veterinary College, University of Prince Edward Island, 550 University Avenue, Charlottetown, PE. C1A 4P3. Canada

## Abstract

Infectious salmon anaemia (ISA) virus (ISAV) is a fish orthomyxovirus that has recently been assigned to the new genus *Isavirus *within the family *Orthomyxoviridae*. It possesses the major functional characteristics of the virus family including haemagglutinating, receptor destroying enzyme (RDE), and fusion activities associated with the virion surface proteins. It is generally accepted that ISAV agglutinates erythrocytes of several fish species and that the ISAV RDE activity dissolves this haemagglutination reaction except for Atlantic salmon (*Salmo salar*) erythrocytes. We used electron microscopy to examine the physical interaction between ISAV and erythrocytes from Atlantic salmon and rainbow trout (*Oncorhynchus mykiss*) during haemagglutination. We present evidence that ISAV enters into Atlantic salmon erythrocytes. Atlantic salmon erythrocytes incubated with ISAV for 4 hours showed endocytosis of the virus particles, which is consistent with virus infection. These observations suggest that the lack of dissolution of ISAV-induced haemagglutination of Atlantic salmon erythrocytes favours virus infection of the erythrocytes. Moreover, such a haemagglutination-infection phenotype is fundamentally different from haemagglutination by avian and mammalian orthomyxoviruses, and is indicative of a different pathogenesis for the fish orthomyxovirus.

## Findings

Infectious salmon anemia (ISA) virus (ISAV) is a fish orthomyxovirus that has recently been assigned to the new genus *Isavirus *within the family *Orthomyxoviridae*. The virus causes a fatal clinical disease in Atlantic salmon with signs of exophthalmia, pale gills, ascites, congestion of gut, enlargement of liver and spleen, petechial hemorrhages in the visceral organs, and severe anemia [1]. In other fish species, ISAV produces asymptomatic infection in experimental challenges, but in rainbow trout, some virulent ISAV strains have been shown to induce mortality suggesting host susceptibility, virus pathogenicity and dose as determinants of the outcome for ISAV infection in fish [2].

Enveloped viruses like ISAV enter target cells by attachment to receptor molecules on the plasma membrane and this initial attachment determines the host range and tissue tropism of the virus [3]. Hemagglutinin-esterase (HE) is a major surface glycoprotein of ISAV with dual function: the haemagglutinin portion is used for host cell recognition by attaching to the cellular sialic acid receptor for inducing infection through the endosomal pathway [4] whereas the esterase portion is a receptor destroying enzyme (RDE) that dissolves the haemagglutinin binding thereby allowing release of new virus particles from infected cells [5]. Infectious salmon anemia virus has been shown to agglutinate erythrocytes of several fish species except brown trout (*Salmo trutta*) and the RDE activity allowed the virus to elute from erythrocytes of other fish species except Atlantic salmon, which did not elute after 24 hours [6]. This has been suggested to be related to the pathogenicity of the virus in that viruses that were able to elute also caused low mortality in a challenge experiment [7]. In the present study, we performed haemagglutination tests using Atlantic salmon and rainbow trout erythrocytes with two ISAV strains, NBISA01 and RPC/NB 04-0851, of differing genotypes and pathogenicity phenotypes. NBISA01 is a highly pathogenic strain belonging to the North American genotype, whereas RPC/NB 04-085-1 is a low pathogenic strain of the European genotype found in eastern Canada and its HE protein places it in a unique highly polymorphic region (HPR) group [2]. At specific intervals the haemagglutination reactions were sampled and processed for electron microscopy to visualize the physical relationships between the virus and the erythrocytes. Duplicate samples were used to quantitate the viral mRNA levels by real-time RT-PCR in order to confirm presence of virus in the samples and detect any virus replication.

The erythrocytes used for haemagglutination in this study were collected from specific pathogen free Atlantic salmon and rainbow trout weighing 20–30 g. The ISAV strains used were grown in TO cell line as previously described [2]. The haemagglutination reaction was carried out with 50 μl of the 10^9.75 ^TCID_50_/ml for NBISA01 or 10^4.25^TCID_50_/ml for RPC/NB 04-085-1 and 50 μl of 1% erythrocytes of Atlantic salmon or rainbow trout in a microhemagglutination plate, according to the procedure described by Falk *et al*. [6]. In order to obtain adequate volumes of samples for electron microscopy and real time RT-PCR, the reaction volumes were scaled up in 48-well tissue culture plates. The haemagglutination reactions were incubated at room temperature (24°C) and samples were collected at 0.5, 1.5, 4, 18, and 36 hours and processed for electron microscopy.

To process the samples for electron microscopy, the haemagglutination reactions from each well were pooled and fixed overnight at 4°C in 3% final glutaraldehyde solution in 0.1 M phosphate buffer. The pellets were recovered by centrifugation at 3,000 rpm for 10 minutes and resuspended in 1 ml of 0.1 M phosphate buffer. The samples were centrifuged at 4,000 rpm for 10 minutes twice to completely remove the glutaraldehyde. Secondary fixation was done on the pellet using 1 ml of 1 % osmium tetra-oxide in 0.1 M phosphate buffer for 30 minutes. The fixed samples were centrifuged at 4,000 rpm for 10 minutes and the pellet was embedded with 4% agar in distilled water and cut into pieces before dehydration in ascending concentrations of ethanol (50, 70, and 95% and absolute ethanol). A 5-minute propylene oxide step was done before the infiltration procedure. Infiltration was carried out with a mixture of epon resin and propylene oxide in a ratio of 1:1 and 1:3 each for 30 minutes, and a final overnight infiltration step with 100% epon in vacuum. The pellet pieces were then embedded in epon resin overnight at 60°C. Semi-thin sections (0.5 μm) were cut from two pieces of pellet from each experimental unit and stained with 1% toluidene blue in 1% sodium tetra-borate solution and viewed under light microscope. Ultrathin sections (80 nm) were cut and recovered using copper super grids and double stained with uranyl acetate and Sato's lead stain. The sections were examined using a Hitachi H7500 transmission electron microscope operated at 80 KV. The experimental out put presented in here is a result of replicate observations.

Infectious salmon anemia virus real-time RT-PCR was done using the LightCycler 1.2 system with RNA Amplification Kit SYBR Green I (Roche Applied Science) and PCR primers FA-3/RA-3 targeting a 220-bp product on ISAV segment 8 [8]. For this, the pooled haemagglutination reaction samples collected at 0, 18, and 36 hours were centrifuged at 10,000 rpm for 5 minutes in a microfuge to pellet the erythrocytes and total RNA was then extracted using TRIZOL reagent (Invitrogen Life Technologies) and the RNA pellet was dissolved in 15 μl of RNase free water of which 1 μl was used in real-time RT-PCR. The 20 μl-reaction volume included 19 μl of the master mix containing 0.3 μM each of the forward and reverse primers, 4 μl SYBR Green, 0.2 μl LC-RT-PCR enzyme mix, 3 μl resolution solution, 0.005 μM MgCl_2_, and 9.4 μl of nuclease-free water. The thermal conditions were one cycle of reverse transcription at 55°C for 30 min, initial denaturation at 95°C for 30s followed by 50 cycles of 95°C for 5s, 59°C for 10s, 72°C for 10s, and data acquisition at 80°C for 2s. Melting curve analysis was performed from 70°C to 95°C in 0.1°C/s increments to assess the specificity of the RT-PCR products. The quantitative (Ct values) and melting curve data were analyzed using Light Cycler software version 3.5 (Roche Applied Science). The real-time reaction products were also resolved by 1% agarose gel electrophoresis in 0.5 × TBE buffer and stained with ethidium bromide and photographed under 304 nm UV light.

**Figure 1:**
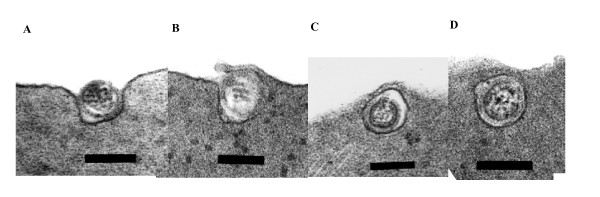
**Transmission electron micrographs reveal the various stages of apparent endocytosis of the NBISA01 virus in Atlantic salmon erythrocytes**. **(A) **a virus particle within an invagination of the plasma membrane (bar = 139 nm); **(B) **partial closure of the pit containing a virus particle (bar = 139 nm); **(C) **and **(D) **virus particle with in a vesicle in the erythrocyte cytoplasm (bar = 139 nm).

Transmission electron microscopic analysis of the ultrathin sections showed the NBISA01 virus closely apposed to the cell membrane of Atlantic salmon erythrocytes and the apparent stages of endocytosis where there was an initial close apposition of the virus, the presence of the virus within an invagination of the plasma membrane that has formed a pit, partial closure of the pit, and virus particles in membrane bound vesicles within the cytoplasm of erythrocytes by 4 hours (Figure 1). In the 18-hour sample, we observed stages of endocytosis of NBISA01 in Atlantic salmon erythrocytes as well as a number of virus particles within vesicles in the cytoplasm. Unlike with the Atlantic salmon erythrocytes, it was possible to locate only one intracellular virus particle in the rainbow trout erythrocytes in either the 4-hour or 18-hour sample after thorough examination of the samples. Analysis of the haemagglutination tests with RPC/NB 04-085-1 virus showed close apposition of the virus to erythrocytes of both Atlantic salmon and rainbow trout by 1.5 hours but neither close apposition nor entry in the erythrocytes at 4 and 18 hours. In the 4-hour samples we were able to observe invaginations in the erythrocytes' plasma membrane that probably had been induced by previous viral attachment to the erythrocytes.

**Figure 2:**
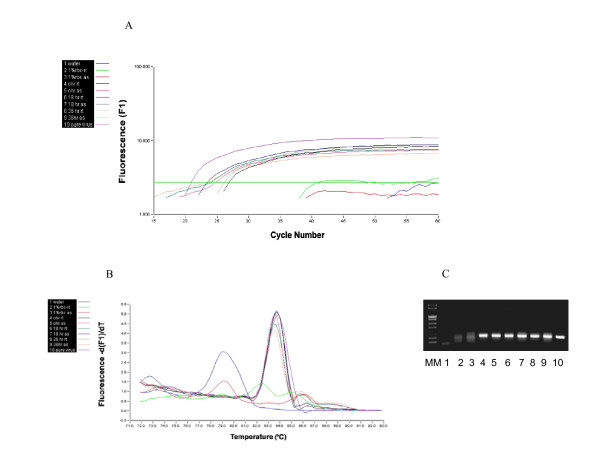
**Amplification, melting curve, and agarose gel electrophoresis of RT-PCR targeting a 220-bp product on ISAV segment 8 using total RNA from haemagglutination tests at different sampling points**. **(A) **Real time RT-PCR amplification curves (water (Ct = 0) – no template negative control; 1% rbc rt (Ct = 0) – rainbow trout erythrocyte control; 1% rbc as(Ct = 0) – Atlantic salmon erythrocyte control; 0 hr rt (Ct = 28.6 ± 4.62) – NBISA01 haemagglutination reaction of 1% rainbow trout erythrocyte sampled at 0 hour; 0 hr as(Ct = 30.8 ± 0.70) – NBISA01 haemagglutination reaction of 1% Atlantic salmon erythrocyte sampled at 0 hour; 18 hr rt (sample not run in triplicate because of insufficient total RNA) – NBISA01 haemagglutination reaction of 1% rainbow trout erythrocyte sampled after 18-hour incubation; 18 hr as (Ct = 32.62 ± 1.10) – NBISA01 haemagglutination reaction of 1% Atlantic salmon erythrocyte sampled after 18-hour incubation; 36 hr rt (Ct = 30.27 ± 0.63) – NBISA01 haemagglutination reaction of 1% rainbow trout erythrocyte sampled after 36-hour incubation; 36 hr as (Ct = 27.59 ± 0.70) – NBISA01 haemagglutination reaction of 1% Atlantic salmon erythrocyte sampled after 36-hour incubation; NBISA01 (Ct = 28.46 ± 0.52) – virus positive control). **(B) **melting curve of the real time RT-PCR of the run in (A). **(C) **PCR products resolved on 1% agarose gel electrophoresis and visualized by ethidium bromide staining. The order of the lanes in the gel picture is the same as in the amplification curves (A).

The lack of haemagglutination elution in the pathogenic ISAV strains has been explained by others as being due to the absence of a functional receptor-destroying enzyme, which allows the virus to persistently attach to erythrocytes [7]. Our observation of NBISA01 virus entry into erythrocytes of Atlantic salmon suggests that the lack of hemagglutination elution in pathogenic ISAV strains in conventional hemmaglutination tests favours virus infection of the erythrocytes. This is the first time such observation is reported and to the best of our knowledge such a haemagglutination-infection phenotype is fundamentally different from haemagglutination by avian and mammalian orthomyxoviruses, and indicates a different pathogenesis for the fish orthomyxovirus. Earlier publications relate ISAV-induced pathogenesis of the anemia to haemagglutination of erythrocytes by the virus followed by uptake of the virus-coated erythrocytes by the immune cells [9]. However, our present observation of the highly pathogenic NBISA01 strain inside Atlantic salmon erythrocytes, and the nucleated nature of fish erythrocytes lead us to suggest that the ISAV-induced anemia may be linked to entry and possible multiplication of pathogenic virus in erythrocytes. To test this hypothesis, we used real-time RT-PCR to assess the viral mRNA levels in the haemagglutination reaction samples taken at 18 and 36 hours. The real-time RT-PCR data are presented in Figure 2. Figure 2A shows that there was a slight increase in viral mRNA transcripts by 36 hours in the haemagglutination reaction of Atlantic salmon erythrocytes with NBISA01 virus, which is indicated by a lower mean Ct value (27.59 ± 0.70) compared to the 0-hour (30.8 ± 0.70) and 18-hour samples (32.62 ± 1.10). This increase may be indicative of an early virus replication; however, a longer sampling interval will be necessary to confirm if ISAV replication occurs in fish erythrocytes. Figure 2B shows a single fluorescence peak on analysis of the melting curve, and Figure 2C shows the 220 bp product by agarose gel electrophoresis, indicating that the amplifications were virus-specific, and that there were uniform virus populations in the individual virus samples.

In a fish challenge experimental study conducted in our lab to investigate correlates of ISAV virulence, the NBISA01 strain was shown to induce very high mortality in Atlantic salmon (95%) and moderate mortality in rainbow trout (50%) [2]. In the present study, the extent of endocytosis of this virus strain in erythrocytes of the two different hosts seems to be related to host susceptibility to the virus in that in Atlantic salmon, which is the susceptible host, we were able to observe a lot of apparent virus endocytosis by 4 hours and intracellular virus particles by 18 hours whereas in rainbow trout, which is a resistant host, there was limited virus endocytosis by 4 hours and none by 18 hours. Consistent with this analysis were our observations with ISAV strain RPC/NB 04-0851. This virus was shown to induce very low mortality in Atlantic salmon (18.2%) and no mortality in rainbow trout [2]. In the present electron microscopy analysis of the haemagglutination test reaction, RPC/NB 04-0851 strain showed close apposition to erythrocytes by 1.5 hours but neither close apposition nor entry in both Atlantic salmon and rainbow trout erythrocytes at 4 and 18 hours. In the 4-hour samples we observed invaginations in the erythrocytes' plasma membrane that probably had been induced by previous viral attachment to the erythrocytes. These results suggest an initial attachment of the virus to erythrocytes and a later release of the virus. This absence of virus endocytosis with the less pathogenic ISAV strain when added to the evidence of haemagglutination-elution in the non-virulent strains [7] highlights a new understanding of ISAV virulence variation among different strains.

In conclusion, these results show apparent endocytosis of the high pathogenic NBISA01 strain into erythrocytes of Atlantic salmon to a larger extent and in rainbow trout to a minor extent. The entry of the virus into erythrocytes is favoured by the lack of haemagglutination elution of the pathogenic ISAV strains and is suggestive of virus infection of Atlantic salmon erythrocytes. The low pathogenic RPC/NB 04-085-1 strain was able to closely appose to erythrocytes of both hosts at an earlier sampling point but there was no morphological evidence of endocytosis by erythrocytes of both hosts at the later sampling point.

## List of abbreviations

Infectious salmon anaemia virus (ISAV), haemagglutination-esterase (HE), receptor-destroying enzyme (RDE), Tris borate EDTA (TBE).

## Competing interests

The author(s) declare that they have no competing interests.

## Authors' contributions

STW conducted all the experiments and wrote the manuscript. DWW and GMW instructed STW in the use of the electron microscope, interpretation of electron micrographs, and edited the paper. MJTK assisted with running the real-time RT-PCR assays. FSBK conceived the study, coordinated the research efforts and edited the paper. All five co-authors read and approved the final manuscript.
